# Primary central nervous system post-transplant lymphoproliferative disorder after allogeneic stem cell transplantation: a case report

**DOI:** 10.3389/fonc.2024.1284577

**Published:** 2024-01-19

**Authors:** Margaret Tugend, Jordan Dattero, Tsiporah Shore, Vlad A. Korben, Raymond F. Sekula

**Affiliations:** ^1^ Department of Neurological Surgery, Columbia University, New York Presbyterian Hospital, New York, NY, United States; ^2^ Department of Brain and Cognitive Sciences, Massachusetts Institute of Technology, Boston, MA, United States; ^3^ Department of Medicine, Division of Hematology/Oncology, Weill Cornell Medicine, New York Presbyterian Hospital, New York, NY, United States; ^4^ Department of Pathology, Columbia University, New York Presbyterian Hospital, New York, NY, United States

**Keywords:** cerebellopontine angle, diffuse large B-cell lymphoma, polyploid, MATRix regimen, double umbilical cord allogeneic transplant, primary central nervous system lymphoma, post-transplant lymphoproliferative disorder, case report

## Abstract

**Purpose:**

Primary central nervous system, diffuse large B-cell lymphoma, post-transplant lymphoproliferative disorder in the cerebellopontine angle after an allogeneic stem cell transplantation has never been reported in the literature. Typically, diffuse large B-cell lymphoma is non-polyploid. We report the first case of a patient with polyploid post-transplant lymphoproliferative disorder in the cerebellopontine angle who presented with back pain.

**Case presentation:**

A 45-year-old man with a history of nodular sclerosing classic Hodgkin lymphoma stage IIB treated with systemic chemotherapy, external radiation and autologous stem cell transplant and double umbilical cord allogeneic transplant presented with several weeks of back pain. He was found to have a small right cerebellopontine angle mass thought to be consistent with a meningioma. Patient presented again two weeks later with acute onset of severe headache, right sided ptosis, right facial numbness, weakness and possible seizure event. Repeat MRI scans showed an interval and significant increase of the right cerebellopontine angle lesion. Biopsy of the cerebellopontine angle lesion was planned with suspicion of lymphoma. Intraoperative pathology consultation findings were not consistent with an acoustic neuroma, meningioma, or epidermoid cyst. Lymphoma could not be definitively identified by intra-operative frozen section. However, it was suspected, and a portion of fresh specimen was submitted for flow cytometry analysis. A near total resection of the tumor and decompression of the brainstem was achieved. Final pathologic analysis was positive for post-transplant lymphoproliferative disorder, monomorphic type, diffuse large B-cell lymphoma, non-germinal center B-cell type, EBV+, post-transplant (allogeneic stem cell) setting (post-transplant lymphoproliferative disorder (PTLD), monomorphic type, diffuse large B-cell lymphoma, non-germinal center B-cell type (non-GCB), EBV-positive under pre-2022 WHO terminology). The patient began a high-dose methotrexate-based regimen (the MATRIX regimen).

**Conclusions:**

Our case illustrates an unusual presentation of post-transplant lymphoproliferative disorder in the cerebellopontine angle in a patient with a remote history of allogeneic stem cell transplantation. It demonstrates the importance of keeping primary central nervous system post-transplant lymphoproliferative disorder on the differential for patients who present with back pain or headache that have a history of allogeneic stem cell transplant.

## 1 Introduction

Lymphoma is a hematological malignancy of the lymphatic system. Diffuse large B-cell lymphoma is the most common type of lymphoma. Although uncommon, lymphomas can present in the central nervous system (CNS). CNS lymphomas are characterized as either primary CNS lymphoma or secondary CNS lymphoma. Primary CNS lymphoma (PCNSL) is an extranodal lymphoma originating in the CNS and constitutes 0.2-2% of lymphomas (1). Patients typically present with progressive symptoms including focal neurological deficits, mental status and behavioral changes, seizures, and signs and symptoms of increased intracranial pressure (headaches, nausea, vomiting, and papilledema) (2). Post-transplant lymphoproliferative disorder (PTLD) is a group of heterogeneous lymphoid proliferations secondary to immunosuppression in transplant recipients. PTLD occurs in 0.5% to 10% of patients depending on transplant type (3). PCNS-PTLD is uncommon (4–9). To our knowledge, there is only one case in the literature of PCNS-PTLD occurring in the cerebellopontine angle (CPA), which was secondary to a kidney transplant (10). For patients younger than 70 years old, chemotherapy and autologous stem cell transplant (SCT) are used to treat CNS lymphoma, with one study reporting a 1-year progression-free survival of 58% and a 2-year overall survival of 46% (11).

Here we report a unique case of PCNS diffuse large B-cell lymphoma (DLBL) PTLD in the CPA, 197 months after a double umbilical cord allogeneic transplant, presenting with back pain, and treated with surgical resection and subsequent chemotherapy.

## 2 Case description

A 45-year-old man with a past medical history of hypothyroidism and nodular sclerosing classic Hodgkin lymphoma stage IIB presented with back pain. Classic Hodgkin lymphoma was treated with systemic chemotherapy, external radiation and autologous stem cell transplant in 2006. Disease relapsed and patient underwent double umbilical cord allogeneic transplant in 2007, which was complicated by numerous infections and graft versus host disease requiring immunosuppression. Patient completed immunosuppression in 2008 and had been in remission since.

Sixteen years after the double umbilical cord allogeneic transplant, the patient presented to an outside hospital, with several weeks of back pain and was treated for suspected lumbar stenosis with steroids. After persistent pain and onset of headache, lumbar and cranial imaging revealed a small right cerebellopontine angle (CPA) mass as well as subarachnoid hemorrhage, trace intraventricular hemorrhage, and some subarachnoid hemorrhage within the spine. Cerebral and spinal angiography demonstrated no evidence of vascular malformation or abnormality. Magnetic resonance imaging (MRI) was obtained and demonstrated intermediate signal on T2 with a hypointense rim and restricted diffusion. The CPA mass was thought to be consistent with a meningioma. The patient was then discharged and was planned for outpatient follow up with neurosurgery.

**Figure 1:**
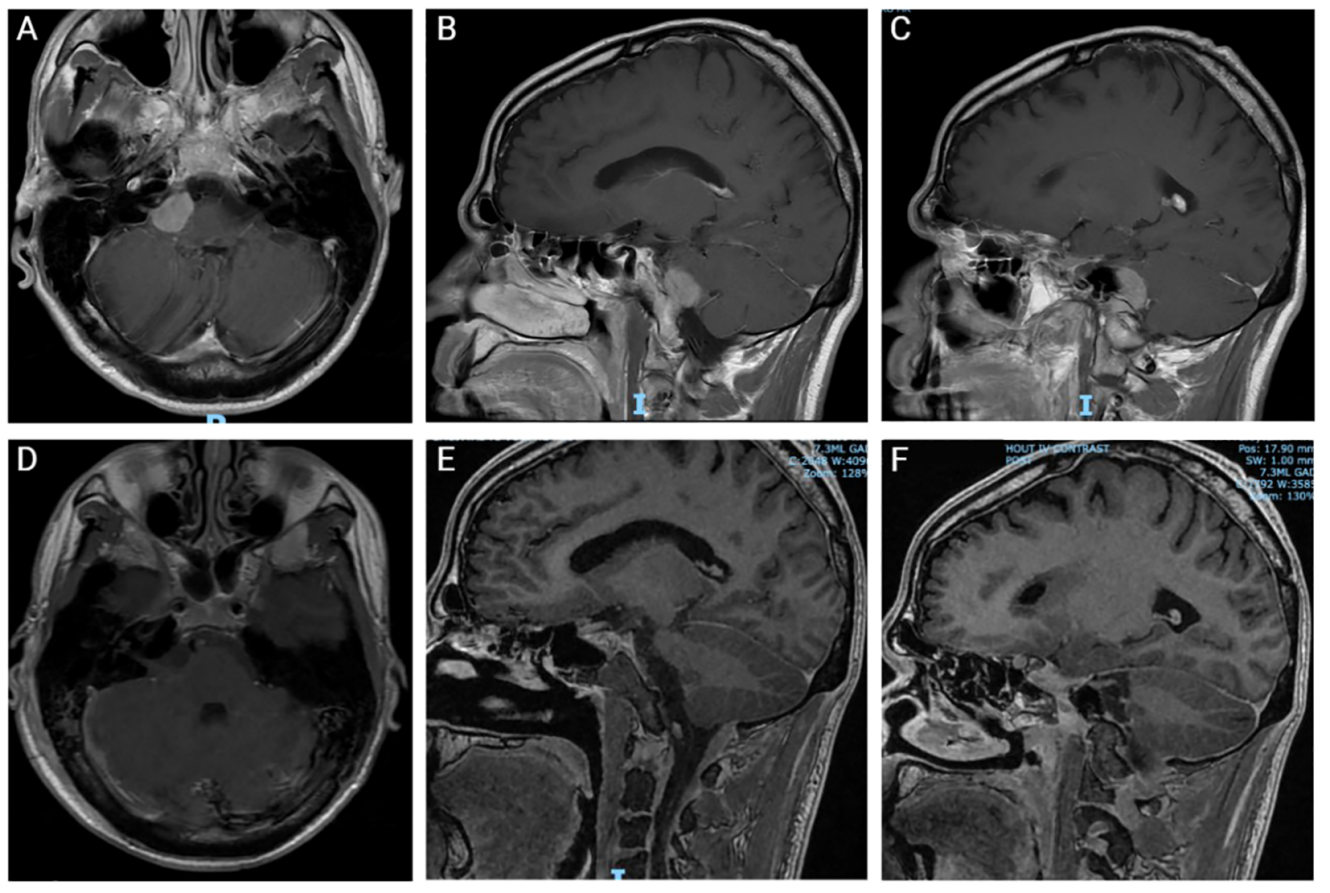
Pre- and post-operative Magnetic Resonance Imaging (MRI). **(A-C)** Preoperative coronal **(A)** and sagittal **(B, C)** MRI with contrast. **(D-F)** Postoperative coronal **(D)** and sagittal **(E, F)** MRI without contrast.

Patient presented again two weeks later with acute onset of severe headache, crushing in quality, affecting the right side of the face, associated with right facial numbness and weakness. Additionally, he reported left sided ptosis. Patient had a possible seizure event with loss of consciousness, no shaking, unclear postictal period, and no tongue laceration or urinary incontinence. The patient received a loading dose of levetiracetam and was started on standing anti-seizure drugs. Repeat MRI scans showed an interval and significant increase of the right CPA lesion (**Figure 1**). Chest, abdomen and pelvis nonenhanced computed tomography (CT) scans were negative for metastatic disease.

Biopsy of the CPA lesion was planned with suspicion of lymphoma. The tumor extended from the tentorium to below the hypoglossal nerve. Intraoperative pathological analysis revealed dense and diffuse infiltrate of large atypical cells with irregular nuclei, multiple small nucleoli, and scant cytoplasm. Findings were not consistent with an acoustic neuroma, meningioma, or epidermoid cyst. Lymphoma was not clearly identified with intraoperative analysis. A near total resection of the tumor and decompression of the brainstem was achieved. The tumor was removed due to clear malignancy and difficulty achieving adequate hemostasis with biopsy alone. A small amount of tumor was left on the facial nerve, which responded with 0.05mA of monopolar stimulation following resection.

**Figure 2:**
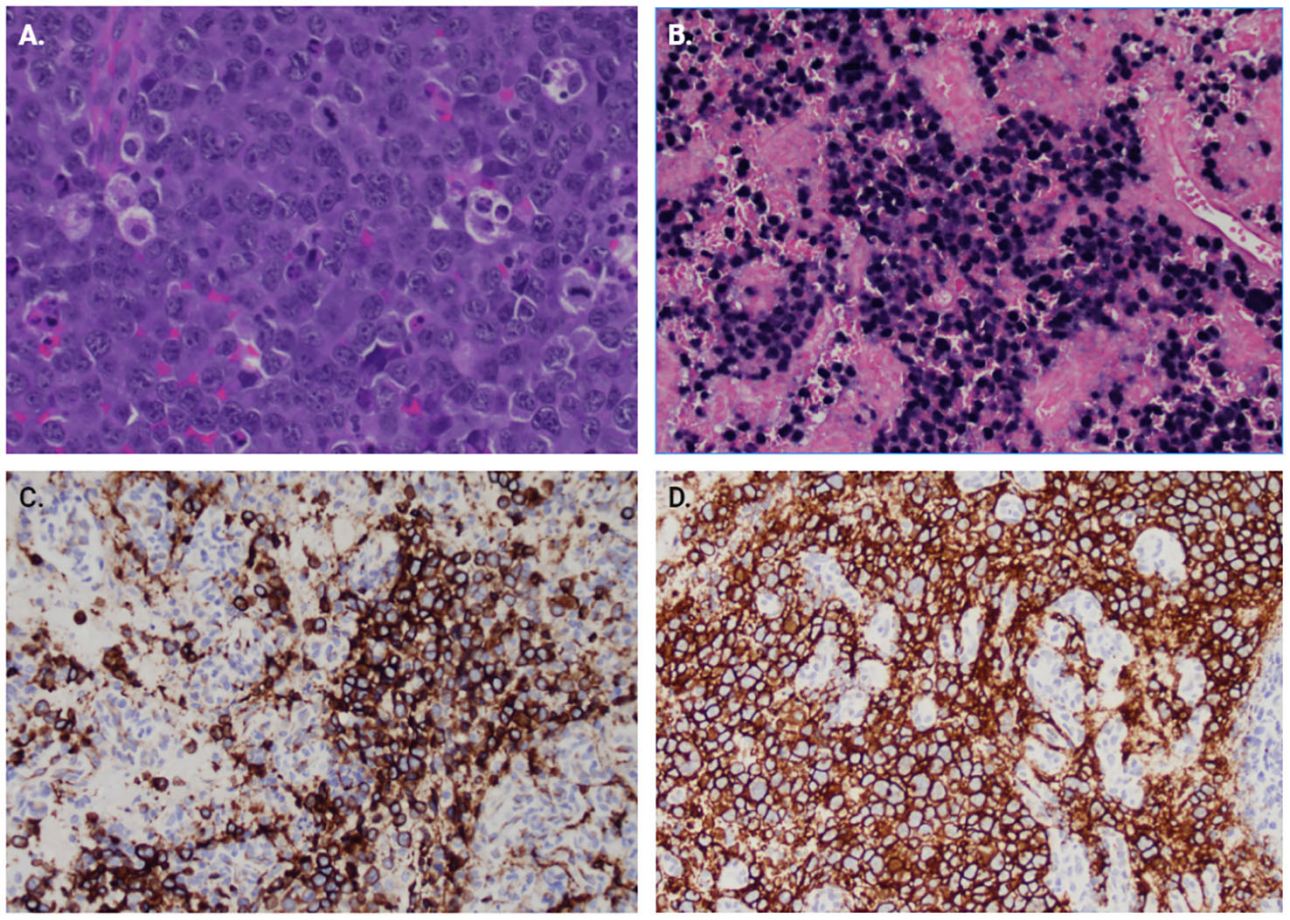
Immunohistochemistry of surgical pathology samples. **(A)** H&E section at 400x showing large atypical cells with irregular nuclei with vesicular chromatin and scant cytoplasm. Multiple mitotic figures are seen. **(B)** EBER-ISH showing positivity for EBV RNA in the majority of tumor cells. Immunostains for B-cell lineage markers, **(C)** CD79a and **(D)** CD20.

As seen in **Figure 2**, the tumor is comprised of large atypical cells with irregular nuclei with vesicular chromatin and scant cytoplasm (A). The tumor cells are positive for B-cell markers CD20 (D)and CD79a (C) and EBER-ISH. The immunohistochemical phenotype of the tumor cells is as follows: CD45+/- (weak, variable), CD20+, CD79a+, CD5-, CD10-, BCL6-, MUM1-, LMO2-, HGAL+, FOXP1-, BCL2+, CD23+, CD43+, MYC+ (80-90%), CyclinD1+/- (40-50%), SOX11-, CD21-, EBER(ish)+. Flow cytometry of the CPA soft tissue showed an aberrant cell population with the following phenotype: CD45dim/-, CD19-, CD20+, cCD79a+, CD22+, CD5-, CD10-, CD23+, CD25+, CD200+, CD30-, CD43+, CD38+, CD33+, HLA-DR+, IgM-/+, IgD-. Final diagnosis was PTLD, monomorphic type, diffuse large B-cell lymphoma (DLBL), non-germinal center B-cell type, EBV+. Cytogenetic results showed no evidence of IGH/BCL2, BCL6, MYC, or CCND1 rearrangement/translocation, however, increased copies of all the genes (IGH, BCL2, BCL6, MYC, and CCND1) in were detected in ~60% cells, indicative of polyploid cells. The patient was sent back to his long-standing oncologist to discuss treatment options for the newly diagnosed primary central nervous system lymphoma. The patient began a high-dose methotrexate-based regimen (the MATRIX regimen).

Five months post-resection, the patient has completed 4 cycles of MATRIX. Brain MRI performed at two months post-resection demonstrated no new nodular or mass-like enhancement to suggest recurrent or increased tumor. Patient underwent an autologous stem cell transplant 4.5 months after resection, conditioned with thiotepa and high-dose carmustine.

## 3 Discussion

Monomorphic DLBCL is the most common histological subtype of PTLD (12). PTLD typically occurs after hematopoietic stem cell transplant or solid organ transplantation. The majority of DLBCL is non-polyploid, with polyploid only accounting for 2.9% of cases. Polyploid DLBCL has been demonstrated to be an independent risk factor for poor prognosis (13). We present a rare case of rapidly enlarging non-immunosuppressed polyploid PTLD-DLBCL in the CPA over 16 years after allogeneic double umbilical cord transplantation.

Previous literature has demonstrated that EBV infection, immunosuppression, age <10 and >60 years old and hematopoietic stem cell transplant are risk factors for PTLD (14). In this case, the patient is 45 years old, was not immunosuppressed, and underwent prior allogeneic stem cell transplantation. PTLD presentation is dependent on the involved organs and rarely presents with lymphadenopathy and classic B symptoms (14). In our case, the patient initially presented with back pain likely secondary to subarachnoid hemorrhage within the spine. His symptoms progressed to headache which resulted in identification of the CPA mass. CPA lymphomas typically present with cranial nerve deficits including, facial weakness, numbness, hearing loss and ataxia (15–17). Although this patient eventually developed facial weakness, he did not initially present with any cranial nerve deficits. Optimal therapies for CNS PTLD have not been established and management is often the same as non-PTLD PCNSL. First line management of non-PTLD primary CNS lymphoma is chemotherapy. MATRix (HD-MTX/cytarabine/thiotepa/rituximab) chemotherapy followed by consolidative high-dose chemotherapy and autologous stem cell transplantation or whole-brain irradiation is associated with a significant improvement in overall survival in DLBCL. However, over one third of patients fail first line treatment and have disease progression (18). Surgical management has typically been reserved for large lesions causing increased intracranial pressure and signs of brain herniation. Despite the dogma that primary CNS lymphoma responds to chemotherapy and radiation, several studies demonstrate that surgical resection may be beneficial in primary CNS lymphoma treatment (19–21). In this case, the patient underwent surgical resection prior to chemotherapy, due to delay in identification of the tumor type as well as bleeding, which has been associated with increased progression free and overall survival (22).

Incidence of PTLD is growing due to the increasing number of transplants and age of recipients (12). Therefore, diagnosing PTLD efficiently is important. In the case presented, diagnosis of PCNS-PTLD was not confirmed until postoperative pathology and cytogenetic analysis. In this case, the patient had rapidly progressive symptoms (headache to facial nerve palsy) in two weeks, making a malignant tumor (e.g. lymphoma) more likely. Differentiating lymphoma from a meningioma on MRI can be challenging. Previous literature has reported that lymphoma ranges from isotense to hypotense on T2 MRI sequences and displays intense post-contrast enhancement (23). Meningiomas range from isointense to slightly hyperintense relative to grey matter on T2 MRI sequences and demonstrate significant post-contrast homogeneous enhancement (24). For future patient care, PCNS-PTLD should be on the differential for patients with aggressive CNS lesions with a history of allogeneic stem cell transplant. PCNS-PTLD in the CPA secondary to allogeneic stem cell transplant, to our knowledge, has never been reported before. Awareness of this case and the outcome may allow physicians to more quickly and accurately diagnose cases of PCNS-PTLD.

This report is limited by the inability to substantiate a causal relationship between allogeneic stem cell transplant and PCNS-PTLD. In this case report, we presume that the PCNS-PTLD is secondary to allogeneic transplant 16 years prior. It is impossible to know whether transplantation was the direct cause of tumor occurrence. A prospective, multicenter, cohort study to evaluate patient outcomes following allogeneic stem cell transplant may provide stronger evidence of a causal relationship between transplantation and developing PCNSL. Additionally, we lack long-term follow-up for this patient, which would allow us to evaluate the efficacy of surgical resection and the MATRIX regimen to treat PCNS PTLD in this case.

## 4 Conclusion

This case report describes a case of primary CNS diffuse large B-cell lymphoma, post-transplant lymphoproliferative disorder in the cerebellopontine angle, 196 months after allogeneic stem cell transplant, treated with surgical resection and subsequent chemotherapy. Our case demonstrates the importance of keeping PCNS-PTLD on the differential for patients who have a history of allogeneic stem cell transplant, even if remote.

## Data availability statement

The original contributions presented in the study are included in the article/supplementary material. Further inquiries can be directed to the corresponding author.

## Ethics statement

Ethical approval was not required for the studies involving humans because IRB approval is not required for individual case reports at Columbia University. See “COLUMBIA UNIVERSITY INSTITUTIONAL REVIEW BOARD/PRIVACY BOARD POLICY: CASE REPORTS”. The studies were conducted in accordance with the local legislation and institutional requirements. The participants provided their written informed consent to participate in this study. Written informed consent was obtained from the individual(s) for the publication of any potentially identifiable images or data included in this article.

## Author contributions

MT: Data curation, Formal analysis, Investigation, Writing – original draft, Writing – review & editing. JD: Data curation, Formal analysis, Writing – original draft, Writing – review & editing. TS: Conceptualization, Investigation, Writing – review & editing. RS: Conceptualization, Investigation, Supervision, Writing – review & editing. VK: Writing – review & editing, Data curation, Formal analysis.
